# Trends and spatial distributions of HIV prevalence in Ethiopia

**DOI:** 10.1186/s40249-019-0594-9

**Published:** 2019-10-17

**Authors:** Getiye Dejenu Kibret, Aster Ferede, Cheru Tesema Leshargie, Fasil Wagnew, Daniel Bekele Ketema, Animut Alebel

**Affiliations:** 1 College of Health Sciences, Debre Markos University, Debre Markos, Ethiopia; 2 College of Health Sciences Department of Nursing, Debre Markos University, Debre Markos, Ethiopia

**Keywords:** Ethiopian demographic and health survey, Cluster, Human immunodeficiency virus, Trend

## Abstract

**Background:**

Globally, by the end of 2018, 37.9 million people were living with human immunodeficiency virus (HIV). Sub-Saharan Africa carries the highest burden with an estimated 71% of the global total. In Ethiopia, an estimated 715 404 people were living with HIV in 2015 and this increased to 722 248 in 2017. This study was to explore the trends and spatial distributions of HIV cases in Ethiopia.

**Methods:**

In this study, we explored the spatial and temporal distribution of persons living with HIV in Ethiopia using data from 2005, 2011, and 2016 Ethiopian Demographic and Health Surveys (EDHS). Geographic information system (Getis-Ord Gi* statistics) and spatial scan statistics (SaTScan) were used for exploratory and confirmatory spatial analyses respectively.

**Results:**

The overall prevalence of HIV in Ethiopia unveiled inconsistent trends, with the majority of areas showing decreasing trends. Hot spot clusters exhibited in all the three surveys, which include areas where Amhara, Afar and Tigray regions share neighbourhoods. In 2005 regionally, Gambella, Addis Ababa, and Harari had the highest prevalence at 6.0, 4.7 and 3.5%, respectively. While in the 2016 survey the highest prevalence (4.8%) was observed in Gambella regional state followed by Addis Ababa (3.4%).

**Conclusions:**

The distribution of HIV infection in Ethiopia is not random in all the three EDHS surveys. High clusters of HIV cases were consistently observed in Addis Ababa and neighbouring areas of the Afar Tigray and Amhara regional states and central Oromia. This analysis revealed that there are still areas which need studying with respect to the epidemic of HIV. In this regard Addis Ababa, certain areas of Amhara regional state, large areas of Afar region and central Oromia require special attention.

## Multilingual abstracts

Please see Additional file 1 for translations of the abstract into the five official working languages of the United Nations.

## Background

Globally, over 76 million people have been infected with human immunodeficiency virus (HIV), and HIV infection has contributed more than 35 million deaths since its emergence. By the end of 2018, about 37.9 million people were living with HIV globally. Sub-Saharan Africa remains among the hardest hit regions by the pandemic, with nearly one in every 25 adults (4.2%) living with HIV, accounting for nearly two-thirds of the global total HIV cases [1, 2].

In Ethiopia, the annual number of HIV infected people showed declining trends since 2002. Over the past two decades HIV prevalence rate decreased from 3.3% in 2000 to 0.9% in 2017, and AIDS-related deaths from 83 000 deaths in 2000 to 15 600 in 2017 [3].

The number of HIV infections among adult Ethiopians was estimated at 722248 in 2017, increasing by 3748 infections from 2016. The highest estimated prevalence’s among adults in 2017 were in Addis Ababa (5%) and Gambella (4%). The two administrative regions, Gambella and Addis Ababa persist with high load of HIV cases for long time so far. This is related to their distinctive characteristics; Gambella region is known for its lower coverage of male circumcision [3] which is one of the risk factors for HIV exposure. Addis Ababa’s high load is related to the trends in distribution that the HIV epidemic in Ethiopia is primarily associated with areas of urban concentration (5.1% in cities above 50 thousand compared to 3.1% in smaller cities and 0.6% in rural areas) [4]. This is related to being a home for high risk groups of population including commercial sex workers, long track drivers and military. On the other hand, the lowest prevalence was estimated to be in the South Nations, Nationalities and People Region (SNNPR) (0.5%) and the Benishangule Gumuz Region (0.6%), signifying the presence of geographical variations [5].

There has been considerable policy and programmatic progress over the past year to support theachievement of the 90–90-90 targets in Ethiopia. Ethiopia’s Federal Ministry of Health launched its Catch-Up-Campaign in 2017 to implement targeted testing in geographic areas where evidence suggests there are large numbers of undiagnosed people living with HIV, with the expectation that it will significantly “catch up” in reaching its treatment targets. Test and treat strategy was adopted nationally and begin implementation in 2017.

In the last two decades, there has been tremendous growth in HIV-related programmes, including education and stigma-reduction programs, behaviour change initiatives, expansion of HIV testing, and programmes aimed at youth. Identifying pockets of caseloads will also help to reveal the uncovered HIV cases [6].

The distribution of HIV prevalence is highly skewed with respect to different socio-economic and geographical areas. Ethiopia is in a state of growing urbanization and scale up of megaprojects that attract a huge work force, composed mostly of youth. These developments result in the emergence of new hotspots and influence the dynamics of HIV transmission. It is therefore indispensable to revitalize and scale up HIV prevention interventions on the basis of geographic and population priorities, involving all the relevant stakeholders [3].

Mapping the spatial patterns can help focus resources for prevention and treatment in the hot spot areas. Identifying high transmission areas and key groups, in combination with the implementation of evidence-based prevention strategies will have the ability to reduce HIV transmissions and achieve substantial epidemic control substantially [7]. A geospatial prioritization with targeted HIV prevention services within and between countries recommended to reduce the burden of HIV in sub-Saharan Africa and have an impact on the global burden of HIV [8].

A cluster analysis of HIV prevalence in Ethiopia from 2011 Ethiopian Demographic and Health Survey (EDHS) showed the prevalence of HIV was in the ranges of 10–21% in certain geographic clusters, particularly in the central, eastern and western parts of the country. It also revealed that the prevalence of HIV was neither randomly nor uniformly distributed in the country, with the epidemic having concentrated in geographic areas of 25 administrative zones in Oromia, Amhara, Tigray, Afar, and Somali regions [9]. These findings necessitate cluster analysis as an important approach to located focus areas. The main objective of this study was to explore the trends and spatial distributions of HIV cases in Ethiopia.

## Methods and materials

### Data source and extraction

The data for this analysis were extracted from EDHS 2005, 2011, and 2016 and accessed from the Measure DHS website (http://www.dhs program.com). It is secondary data analysis from nationwide community-based survey. The data for this analysis were extracted from the 2016 EDHS. The data sets were downloaded in SPSS format with permission from Measure DHS website (http://www.dhs program.com). Data cleaning and recording were carried out in **SPSS** statistical software (IBM SPSS Statistics for Windows, Version 25.0; IBM Corp: Armonk, New York, United States). The HIV related datasets were joined to Global Positioning System (GPS) coordinates of EDHS using the joining variable as recommended by DHS measure.

In the DHS surveys, samples were selected using a stratified, two-stage cluster design, using enumeration areas (EAs) as a primary sampling units and households as the secondary sampling units. The detailed methodology is found in the main report [10].

### Population and outcome measurement

Sampling methods for EDHS populations are described in detail elsewhere [10]. Briefly, all women aged 15 to 49 and men aged 15 to 59 within randomly selected enumeration areas (EAs) were eligible for HIV testing as part of EDHS. HIV infection, the primary outcome in this study, was defined as a confirmed positive HIV test using an ELISA (enzyme-linked immunosorbent assay) algorithm.

### Data analysis

#### Trend analysis

Trends of HIV prevalence by region and residence (urban vs rural) were carried out using excel. A two group *t*-test for the presence of differences between urban and rural prevalence was computed.

#### Spatial analysis

Statistically significant clusters are defined as geographic areas in which the prevalence of disease is higher compared to neighbouring areas. Tests for global clustering detect the existence of at least one cluster, but not the specific location of the cluster(s) [11].

In this study, spatial analysis was performed using the spatial statistics tool in GIS (ESRI ArcGIS Version 10.3; Redlands, California, United States) and Kulldorff’s spatial scan method (SaTScan™ Version 10.3: developed by Martin Kulldorff together with Information Management Services Inc. New York City, United States). SaTScan software has been widely used to detect clustering of areas with high disease prevalence in a variety of health-related fields [11–13].

#### Spatial autocorrelation (global Moran’s *I*)

To detect the presence of clustering in the data, spatial autocorrelation with fixed distance conceptualization was performed in ArcGIS. Spatial autocorrelation based on feature locations and attribute values using the Global Moran’s *I* statistic was calculated and *Z*-scores with corresponding *P*-values were presented. Statistically significant Moran’s *I* index revealed the presence of overall nationwide clustering [14], but it does not show where the actual clustering area is. In this case, local cluster analysis will be required for the confirmation of specific cluster areas.

#### Cluster analysis

Final confirmatory spatial analyses were done using spatial analysis software (SaTScan) and ArcGIS. The SaTScan declares where spatially significant higher rates of aggregates are found. Its output presents the hotspot areas in circular windows, indicating the areas inside the windows are higher than expected distributions compared to the areas outside of the cluster windows. Hot spot areas with high cluster of HIV prevalence and Cold spot areas with low level clusters were identified in ArcGIS.

#### Interpolation

In this study, we used the empirical Bayesian kriging (EBK) technique to predict values for areas where data points were not taken.

Interpolation is based on the assumption that spatially distributed objects are spatially correlated; in other words, things that are close together tend to have similar characteristics [15, 16].

Kriging uses the data twice: the first time to estimate the spatial autocorrelation of the data and the second time to make the predictions to uncover the dependency rules and to make the predictions.

EBK automatically calculates parameters through a process of sub setting and simulations [17]. Other kriging methods calculate the semi variogram from known data locations and use this single semi variogram to make predictions at unknown locations [18]; this process implicitly assumes that the estimated semi variogram is the true semi variogram for the interpolation region. By not taking the uncertainty of semi variogram estimation into account, other kriging methods underestimate the standard errors of prediction [18].

Other kriging models assume that the process follows an overall mean (or specified trend) with individual variations around this mean. Large deviations are pulled back toward the mean, so values never deviate too far. However, EBK does not assume a tendency toward an overall mean, so large deviations are just as likely to get larger as they are to get smaller [16]. Hence, intrinsic random functions inherently correct for trends in the data.

## Results

In the 2005 EDHS, the overall prevalence of HIV was 1.4%. The prevalence among women and men was 1.9 and 0.9% respectively. Regionally, Gambella, Addis Ababa, and Harari had the highest prevalence at 6.0, 4.7 and 3.5%, respectively.

In the 2011 EDHS, the prevalence of HIV was 1.5, 1.9 and 1.0% in the general population, women and men respectively. The highest prevalence was observed in Gambella, Addis Ababa, and Dire Dawa administrative states with 6.5, 5.2 and 4.0%, respectively.

In the third (EDHS, 2016) survey, the overall prevalence of HIV was found to be 0.9%, of which 1.2% among women and 0.6% among men. Regionally, the highest prevalence (4.8%) was observed in Gambella regional state followed by Addis Ababa (3.4%); (Table 1 shows the sample population).

**Table 1:** Sample and frequency distribution of HIV cases in the subsequent EDHS surveys

Survey	Sample	Indeterminate	Tested positive	Percentage
EDHS, 2005	11 374	0	163	1.4
EDHS, 2011	28 532	24	408	1.5
EDHS, 2016	25 776	3	239	0.9

*EDHS* Ethiopian demographic and health surveys, *HIV* Human immunodeficiency virus.

### Trends of HIV

**Fig. 1:**
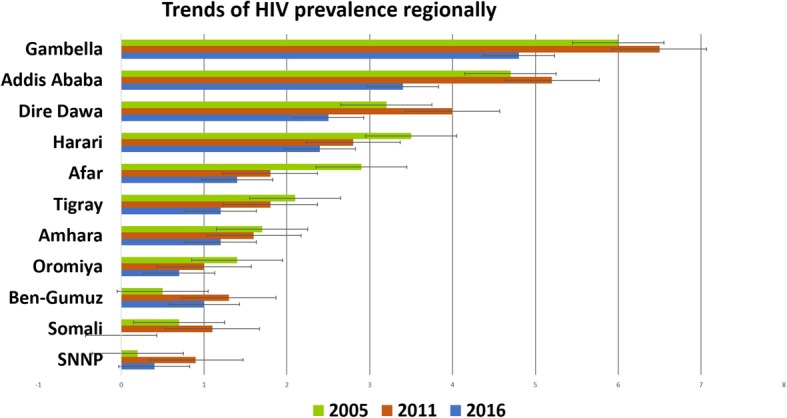
Trends of HIV by region; data from EDHS 2005, 2011, and 2016. HIV: Human immunodeficiency virus

The prevalence of HIV increased from 2005 to 2011 in most of the regions, including Dire Dawa, Addis Ababa, Gambella, South Nations, SNNPR, Benishangule Gumuz, and Somali. On the other hand, in the later 5 years duration, 2011 to 2016, the prevalence was decreased in all of the administrative regions, (Fig. 1).

**Fig. 2:**
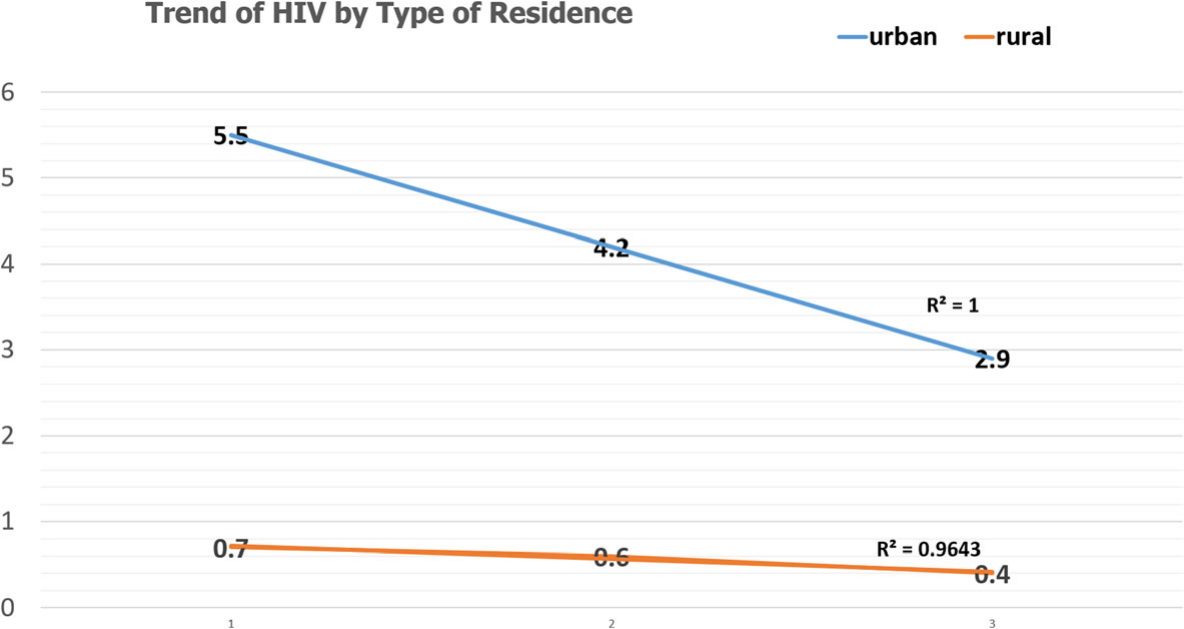
Trends of HIV by Urban and Rural; data from EDHS 2005, 2011, and 2016. *HIV: Human immunodeficiency virus; EDHS: Ethiopia demographic and health surveys*

The prevalence of HIV in urban and rural areas showed decreasing trends, but the decrement in rural is not as steeped as urban areas. There is significant difference between the urban and rural trends (*P* = 0.0086) (Fig. 2).

### Spatial patterns of HIV

The spatial autocorrelation analysis revealed the presence of statistically significant clusters at 0.01, level of significance in each survey (Table 2).

**Table 2:** Spatial autocorrelation analysis of HIV from EDHS (2005, 2011 and 2016)

Survey	Pick clustering distance in meters	Observed Moran’s *I*	Expected Moran’s *I*	*Z*-Score	*P*-value
EDHS, 2005	208 154.00	0.0346	−0.00187	6.65	< 0.01
EDHS, 2011	174 548.95	0.0720	−0.00168	12.57	< 0.01
EDHS, 2016	136 523.40	0.0598	−0.00160	8.75	< 0.01

*EDHS* Ethiopian demographic and health surveys, *HIV* Human immunodeficiency virus.

**Fig. 3:**
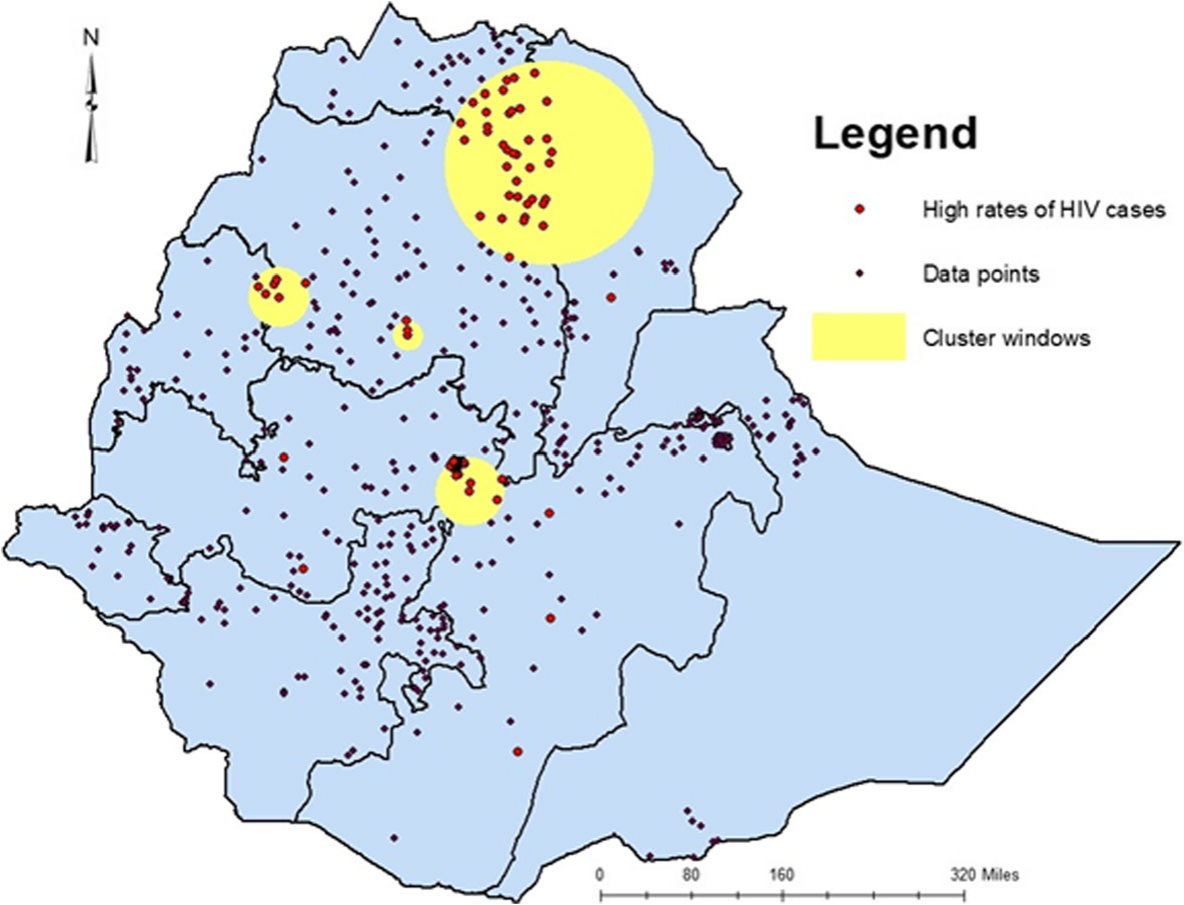
Hot spot clusters of HIV in Ethiopia; in 2005. The rings indicate the statistically significant spatial windows which contain clusters of high HIV cases. HIV: Human immunodeficiency virus

In the 2005 EDHS, areas identified as high clusters of HIV cases include part of Central Tigray, East Tigray, South Tigray, Megale, and Yalo woredas of Afar region, Pawe and Mandura woredas of Benshangul Gumuz region, Dangila woreda of Awi zone, Enarj Enawuga and Goncha sisonesie of East Gojam Zone, North Wollo, Addis Ababa and few parts of Oromia (Fig. 3).

**Fig. 4:**
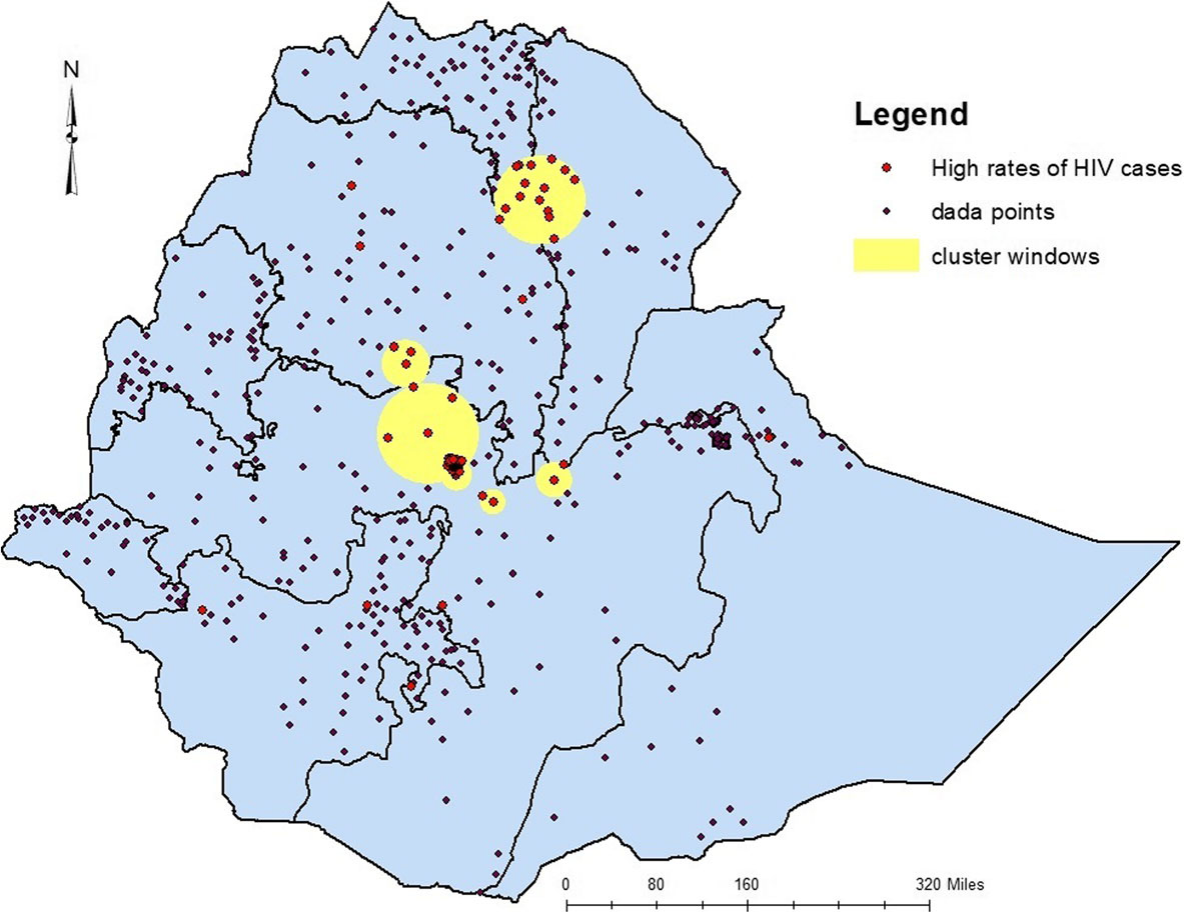
Hot spot clusters of HIV in Ethiopia; in 2011. The rings indicate the statistically significant spatial windows which contain clusters of high HIV cases HIV: Human immunodeficiency virus;

In the 2011 EDHS, areas within the hotspot cluster windows include, almost all parts of Addis Ababa, North Wello zone, Dera district of Suoth Gondar zone and Dembia district of North Gondar zone from Amhara region, few parts of Tigray, few parts of Afar, most parts of central Oromia and few areas of SNNPR. On the other hand, almost all Zonal towns of Amhara regional state, Addis Ababa and Adama City were with high rates of HIV cases (Fig. 4).

**Fig. 5:**
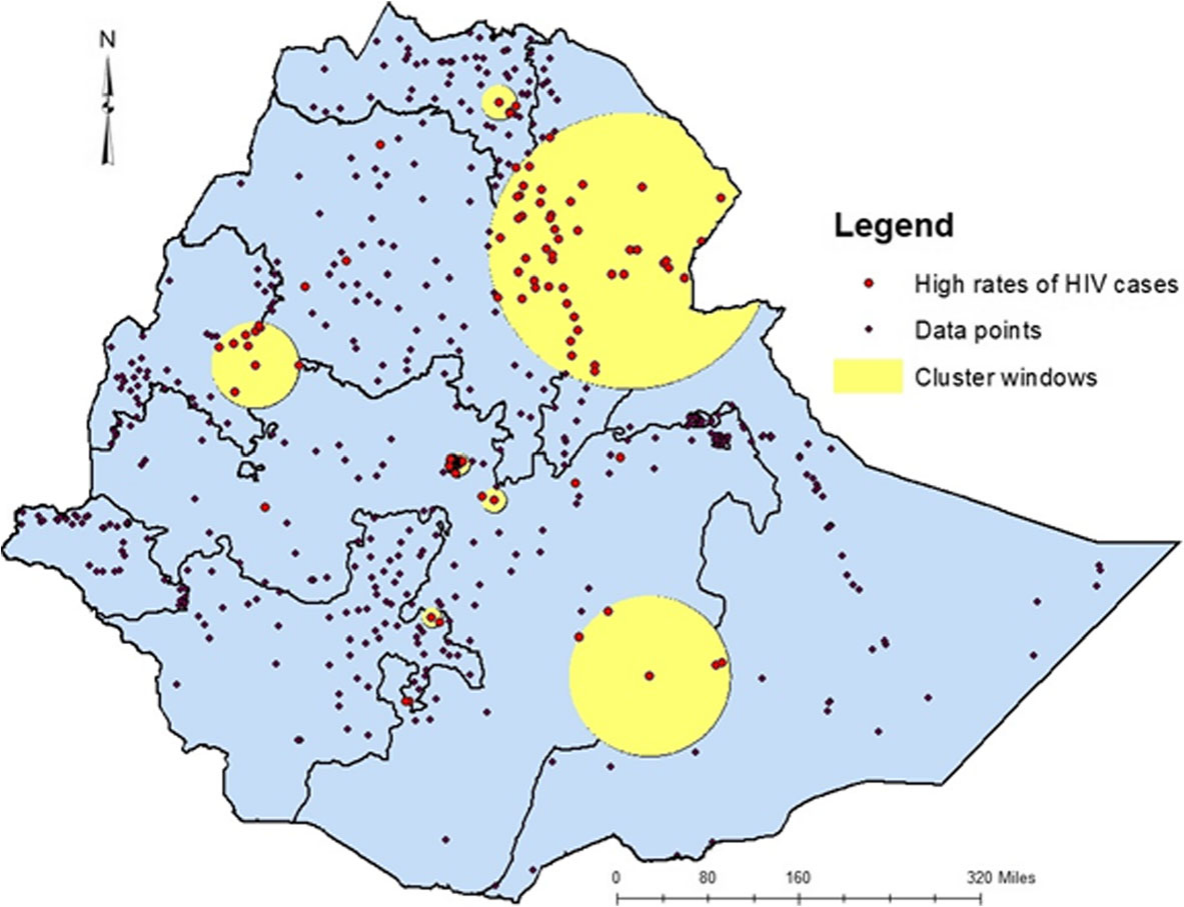
Hot spot clusters of HIV in Ethiopia; in 2016. The rings indicate the statistically significant spatial windows which contain clusters of high HIV cases. HIV: Human immunodeficiency virus

Whereas in the 2016 EDHS, South Wello zone, North Wello zone and Bati district of Amhara regional state, almost all Zones of Afar region (zone1, zone 4 and zone 5), few parts of Tigray (south Tigray and central Tigray), parts of Oromia (East showa, Bale, Borena and west Hararge), few parts of Benshangul-Gumuz (Metekel zone and Kamashi zone) were mapped with significantly high clusters of PLHIV. Several Zonal towns of Amhara region, Addis Ababa, Adama, Jima, Hawassa, Harar, Dire Dawa, and Jigjiga were also identified as high-prevalence urban areas for HIV (Fig. 5).

**Fig. 6:**
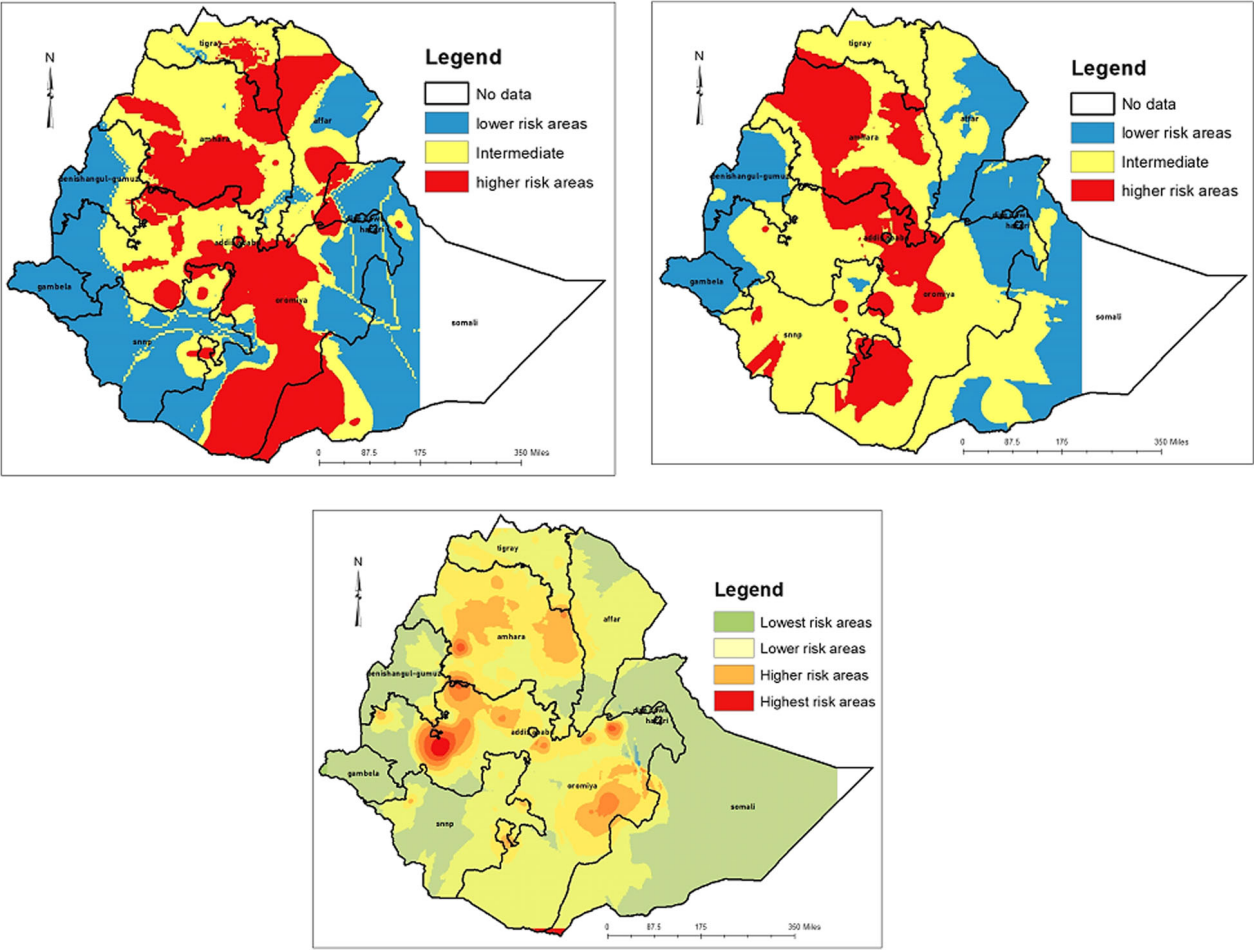
Interpolated spatial trends of HIV in Ethiopia; in 2005 (upper left panel), 2011 (upper right panel) and 2016 (lower panel). The dark red colour indicates the predicted high-risk areas of HIV, yellow ramp colour indicates intermediate risk areas and blue colour indicates less risk areas of HIV. HIV: Human immunodeficiency virus

The empirical Bayesian kriging output mapped the estimated distributions of HIV interpolating the available data to the areas where data were not taken. The estimated high-risk areas in 2005 EDHD were Central, Southwest and Northeastern Amhara, Central, west-central and South Oromia, Southwest Somali and areas where Tigray, Amhara and Afar regions are neighboured (Fig. 6).

## Discussion

The overall prevalence of HIV in Ethiopia were found to be 1.4, 1.5 and 0.9 in 2005, 2011 and 2016 respectively. These figures are lower than the East and Southern Africa regional estimate of 2016 (7%).

The prevalence is higher among female population, 1.9% in 2005 and 2011 and 1.2 in the 2016 EDHS, but this is not statistically significant (*P* = 0.20). However, these figures are lower than some African countries; Zambia 2014, 15.1% [19] and Rwanda 2015, 3.6% [20]. This can be related to the fact that Ethiopia has been implementing different HIV-related programmes, including education and stigma-reduction programs, behaviour change initiatives, expansion of HIV testing, and programmes aimed at youth. The ART - coverage in Ethiopia also reached 62.3%, which is above the sub-Saharan African regional average (53%) [3]. On the other hand, it is higher than the prevalence observed in India 2016, 0.2% [21] and Niger 2012, 0.4 [22]. This alarms that the country still needs more effort to reduce the HIV load below the current figure.

The trends of high HIV clusters in 2005 were in the northeastern parts of Amhara region (bordering with Afar and Tigray regions), South Amhara, south eastern Tigray, north western Afar, Addis Ababa and central Oromia surrounding Addis Ababa. In the 2011 survey the clusters shifted to a few parts of northern Amhara, south eastern Amhara, few parts of south eastern Tigray, few areas of west Afar Addis Ababa and Central Oromia. In the last (EDHD, 2016) survey, the hot spot areas had expanded to almost all parts of Afar regional state, south eastern Oromia eastern parts of Benshangul Gumuz Region and few parts of SNNPR. Hot spot clusters exhibited in all the three surveys include areas where Amhara, Afar and Tigray regions share a neighbourhood. These trend shifts might be related to the inconsistent interventions and program implementations. Most of the HIV intervention programs are donor driven and the effectiveness of the programs highly depend on the local implementers’ strength in appropriately utilizing resources and implementing interventions to the local context.

In the 2011 EDHS, areas identified with an estimated higher risk of HIV were West-central and Northwest Amhara, areas where South Amhara and Central Oromia neighboured, Central and Southern Oromia and a few parts of Southwestern of SNNPR. In the 2016 EDHS, the estimated high-risk cluster areas were highly reduced, and were in few parts of Southwestern Amhara, Northwestern and Southeastern Oromia.

In 2005 EDHS, Cities and Towns in the high-risk cluster windows were Mekele, Woldiya, Addis Ababa and Adama. In 2011, almost all Towns of Amhara regional state, Addis Ababa and Adama and in 2016, almost all Towns of Amhara and Afar regional states, Towns in the South Eastern of Tigray, Addis Ababa and Towns of Oromia regional state surrounding Addis Ababa were in the high-risk cluster windows.

This finding is in line with findings of district level prevalence and geospatial mapping of HIV in South Africa, Nigeria, Mozambique, Burkina Faso, and Malawi which revealed significant variability with hotspots clustered around truck stops and main transport routes [23–26]. This suggests that more attention is needed in the towns and cities within the cluster windows.

The overall prevalence of HIV in Ethiopia unveiled inconsistent trends, with the majority of areas showing decreasing trends. This might be due to the low ART drug coverage and low adherence resulting in high mortality of people living with HIV. The burden still disproportionately affects women. This is in line with evidences reflecting that a range of factors contribute to the peculiar vulnerability of women to the virus. Women are victims of discrimination in the economic, social and political life of the community which factors may directly or indirectly contribute to their exposure to HIV/AIDS. Many of them are also subjected to violence of different kinds ranging from sexual violence to harmful traditional practices which increase their chance of HIV infection [27]. In addition women are said to be biologically more vulnerable to acquire the infection due to the fact that infected semen remains in the vaginal canal for a relatively longer period of time, the exposed mucosal surface area is large and vagina is more susceptible to small tears [28, 29].

Some administrative regions experienced increased HIV prevalence in the years between 2005 and 2011. Still few regional states had an increased prevalence compared to the 2005 figures, which represents a credit to public health prevention programs.

However, this study did not analyse for the contributing factors for the spatial variations in HIV cases which could enable to identify more detailed intervention targets and strategies.

## Conclusions

The trend of HIV prevalence is not consistently decreasing or increasing. In the 2011 survey the magnitude of HIV was increased in most of the regional areas compared to the 2005 survey. Then the magnitude show decreasing trend in 2016 compared the 2011 survey.

The spatial distribution of HIV cases in all the three surveys was not random. Some parts of Amhara regional state, a large area of Afar a few parts of Tigray, Addis Ababa and areas surrounding Addis Ababa continue to show the highest prevalence of HIV. Special attention and evidence-based interventions including HIV/AIDS awareness campaigns, ART services with adherence supports and prevention of mother-to-child transmission of HIV should be targeted in areas identified with hotspot clusters. Investing on reproductive age women means; saving the lives of foetus, new-borns, families and the community at large. In this regard, designing appropriate intervention strategies to protect women of reproductive ages should be a prime concern of the Ethiopian government and other stakeholders.

## Supplementary information

**Additional file 1.** Multilingual abstracts in the five official working languages of the United Nations

## Data Availability

All the data supporting the study findings are within the manuscript. Additional detailed information and raw data will be shared upon request addressed to the corresponding author.

## Abbreviations

AIDS: Acquired immune deficiency syndrome
DHS: Demographic and health surveys
EAs: Enumeration areas
EBK: Empirical bayesian statistics
EDHS: Ethiopia demographic and health surveys
GIS: Geographic information system
GPS: Geographic positioning system
HIV: Human immunodeficiency virus
SaTScan: Spatial Scan Statistics
SNNPR: South Nations, Nationalities and People Region
