# Supplementary material for: Trends and spatial distributions of HIV prevalence in Ethiopia
Source: Infect Dis Poverty. 2019 Oct 17;8:90. doi: 10.1186/s40249-019-0594-9 (PMC6796490; doi:10.1186/s40249-019-0594-9)
Supplement: Supplementary file 1 — Additional file 1. Multilingual abstracts in the five official working languages of the United Nations [file 40249_2019_594_MOESM1_ESM.pdf]

Translation of the abstract into the five official working languages of the United Nations

مسارات انتشار فيروس نقص المناعة البشرية في إثيوبيا وتوزعاته المكانية فيها

جيتي ديجينو كيبرت، أستر فيريد، تشيرو تيسيما، فاسيل واجنيو، دانييل بيكيلي، أنيموت أليبيل.

ملخص
<معلومات أساسية: سيكون هناك سبعة وثلاثين مليون شخص مصاب بفيروس نقص المناعة البشرية (إتش آي في)على مستوى العالم بحلول نهاية عام 2018. تتحمل البلدان الأفريقية جنوب الصحراء الكبرى العبء الأكبر بحوالي 71% من الإجمالي العالمي. ما يقدر بنحو 715404 شخص مصابين بفيروس نقص المناعة البشرية في أثيوبيا في عام 2015، وارتفع هذا العدد إلى 722248 شخص في عام 2017. كانت هذه الدراسة لاستكشاف المسارات والتوزعات المكانية للحالات المصابة بفيروس نقص المناعة البشرية في إثيوبيا.
الطرق: في هذه الدراسة، قمنا بتقصي التوزع المكاني والزماني للأشخاص المصابين بفيروس نقص المناعة البشرية في إثيوبيا باستخدام بيانات الدراسات الاستقصائية الديموغرافية والصحية الإثيوبية (EDHS) لعام 2005 و2011 و2016. استُخدم نظام المعلومات الجغرافية (إحصائيات Getis-Ord Gi *) وإحصاءات المسح المكاني (SaTScan) للتحليلات المكانية الاستكشافية والتأكيدية على التوالي.
<النتائج: كشف انتشار فيروس نقص المناعة البشرية بشكل عام في إثيوبيا عمومًا عن اتجاهات متضاربة، حيث أظهرت غالبية المناطق اتجاهات متناقصة. ظهرت تجمعات البؤر الساخنة في جميع الدراسات الثلاث، والتي شملت المناطق التي تتقاسمها مناطق أمهرة وعفار وتيغري.
الاستنتاجات:إن توزع العدوى بفيروس نقص المناعة البشرية في إثيوبيا ليس عشوائيًا في جميع الدراسات الثلاث للـ EDHS. وقد لوحظت باستمرار تجمعات كبيرة من حالات الإصابة بفيروس نقص المناعة البشرية في أديس أبابا والمناطق المجاورة لولايات عفار وتيغري وأمهرة الإقليمية وفي وسط أوروميا. وأظهر هذا التحليل أنه لا تزال هناك مناطق تحتاج إلى دراسة فيما يتعلق بوباء فيروس نقص المناعة البشرية. في هذا الصدد، تحتاج أديس أبابا، وبعض المناطق في ولاية أمهرة الإقليمية، ومناطق كبيرة من إقليم عفار ووسط أوروميا، إلى اهتمام خاص.

Translated from English version into Arabic by Amouna Ahmat Oumar, Revised by Samah Almoghrabi, through

TRANSLATORS
WITHOUT BORDERS

埃塞俄比亚 HIV 的流行趋势和空间分布

Getiye Dejenu Kibret, Aster Ferede, Cheru Tesema, Fasil Wagnew, Daniel Bekele and Animut Alebel

摘要
引言：截至 2018 年底，全球有 3790 万人类免疫缺陷病毒（HIV）患者。其中，撒哈拉以南非洲的 HIV 疾病负担最重，约占全球总数的 71%。2015 年，埃塞俄比亚有 715 404 例患者，2017 年增加至 722 248 人。该研究旨在探讨埃塞俄比亚 HIV 病例的流行趋势和空间分布。

方法：在本研究中，我们使用 2005 年、2011 年和 2016 年埃塞俄比亚人口与健康调查（EDHS）的数据，探讨了埃塞俄比亚 HIV 患者的时空分布。地理信息系统（Getis-Ord Gi* statistics）和空间扫描统计（SaTScan）分别用于探索和确证空间分析。
结果：在埃塞俄比亚，HIV 在不同地区的流行趋势有所不同，但是大多数地区呈下降趋势。三次调查均纳入所以热点地区，包括具有 Amhara，Afar 和 Tigray 等相邻地区。
结论：在三次 EDHS 调查中，埃塞俄比亚的 HIV 病例数并非随机分布。在 Addis Ababa 以及 Afar Tigray、Amhara 地区和 Oromia 中部邻近地区发现了大量的 HIV 病例。该研究表明，仍需要对一些地区的 HIV 流行情况进行研究，尤其是要特别关注 Addis Ababa、Amhara 地区的一些区域、Afar 地区的大部分区域以及 Oromia 中部地区。

Translated from English version into Chinese by Cong-Shan Liu, edited by Jin Chen

CHINESE CENTER FOR DISEASE CONTROL AND PREVENTION
NATIONAL INSTITUTE OF PARASITIC DISEASES

Tendances et répartition spatiale de la prévalence du VIH en Éthiopie

Getiye Dejenu Kibret, Aster Ferede, Cheru Tesema, Fasil Wagnew, Daniel Bekele et Animut Alebel

Résumé
Contexte : À la fin de 2018, 37.9 millions de personnes étaient porteuses du virus de l'immunodéficience humain (VIH)dans le monde. C'est l'Afrique subsaharienne qui en supporte le plus lourd fardeau avec environ 71 % du total mondial. En Éthiopie, on estime que 715 404 personnes étaient infectées en 2015 et 722 248 en 2017. Le but de cette étude était d'étudier les tendances et la répartition spatiale des cas de VIH en Éthiopie.
Méthodes : dans cette étude, nous avons exploré la répartition spatiale et temporelle des personnes séropositives au VIH en Éthiopie à l'aide des données de 2005, 2011 et 2016 des enquêtes démographiques et sanitaires éthiopiennes (EDHS). Un système d'information géographique (statistiques Getis-Ord Gi*) et des statistiques d'analyse spatiale (SATScan) ont été utilisés respectivement pour des analyses spatiales exploratoires et confirmatoires.
Résultats : La prévalence globale du VIH en Éthiopie a révélé des tendances incohérentes, la majorité des zones affichant des tendances décroissantes. Les groupes de points chauds révélés dans les trois enquêtes incluaient des zones de quartiers partagés entre les régions d'Amhara, d'Afar et du Tigré.
Conclusions : La distribution de l'infection par le VIH en Éthiopie n'est aléatoire dans aucune des trois enquêtes EDHS. Des regroupements importants de cas d'infection ont été constamment observés à Addis-Abeba et dans les régions voisines des états régionaux d'Afar, du Tigré et d'Amhara et d'Oromia centrale. Cette analyse a révélé qu'il existe toujours des zones qui doivent être étudiées en ce qui concerne l'épidémie de VIH. À cet égard, Addis-Abeba, certaines zones de l'état régional d'Amhara, des vastes zones de la région d'Afar et de l'Oromia centrale demandent une attention particulière.

Translated from English version into French by Suzanne Assenat, Revised by Eric Ragu, through

Тенденции и пространственно - временное распределение заболеваемости ВИЧ в Эфиопии

Гети Деену Кибрет, Астер Фереди, Черу Тесема, Фасил Вагнев, Дэниел Бекель и Анимут Альбел

Аннотация

Справочная информация: К концу 2018 года вирус иммунодефицита человека (ВИЧ) был обнаружен у 37.9 миллионов жителей по всему миру. Наибольшая заболеваемость отмечена в Африке к югу от Сахары; она составляет примерно 71% от общемировой. Согласно статистике, в 2015 году 715 404 человека заразились ВИЧ в Эфиопии, а в 2017 году этот показатель увеличился до 722 248 человек. Данное исследование было проведено с целью изучения тенденций и пространственного распределения заражения ВИЧ в Эфиопии.

Методы: Мы изучали пространственно-временное распределение заражённого населения в Эфиопии, используя данные Эфиопских демографических и медицинских обследований (ЭДМО) за 2005, 2011 и 2016 годы. В рамках исследования мы использовали географическую информационную систему (статистика Getis-Ord Gi *) и статистику пространственного сканирования (SaTScan) для подтверждения полученных результатов

Результаты: Общая распространенность ВИЧ в Эфиопии демонстрирует противоречивые тенденции: в большей части страны наблюдается снижение показателей. Кластеры «горячих точек» представлены во всех трех исследованиях, включая районы, где Амхара, Афар и Тиграй соседствуют друг с другом.

Выводы: Исследование показало, что распространение ВИЧ-инфекции на территории Эфиопии вызвано не случайно. Частые случаи заражения ВИЧ наблюдаются в Аддис-Абебе и его соседних районах, в Афар-Тиграй, в государствах региона Амхара и центральной Оромии. Проведённый анализ показал, что заболеваемость ВИЧ ещё недостаточно хорошо изучена. В связи с этим, Аддис-Абеба, некоторые районы Амхара, крупные районы Афарского региона и центральной Оромии требуют особого внимания.

Translated from English version into Russian by Anna Kukharchuk, Revised by Lamiya Mehtieva, through

Las tendencias y la distribución espacial de la prevalencia del VIH en Etiopía

Getiye Dejenu Kibret, Aster Ferede, Cheru Tesema, Fasil Wagnew, Daniel Bekele y Animut Alebel

Resumen

Antecedentes: a nivel mundial, a finales de 2018, 37.9 millones de personas vivían con el virus de la inmunodeficiencia humana (VIH). El África subsahariana es la región con la mayor carga, con un estimado del 71 % del total mundial. En Etiopía, se estima que 715 404 personas vivían con el VIH en 2015, y esta cifra aumentó a 722 248 en 2017. Este estudio se realizó para explorar las tendencias y la distribución espacial de los casos de VIH en Etiopía.

Métodos: en el presente estudio, examinamos la distribución espacial y temporal de las personas que viven con el VIH en Etiopía, utilizando datos de las Encuestas Sanitarias y Demográficas de Etiopía (EDHS, por sus siglas en inglés) de los años 2005, 2011 y 2016. El sistema de información geográfica (Getis-ord Gi* statistics) y las estadísticas de exploración espacial (SaTScan) se utilizaron para los análisis espaciales exploratorios y confirmatorios, respectivamente.

Resultados: la prevalencia general del VIH en Etiopía reveló tendencias incongruentes, y la mayoría de las zonas mostraron tendencias decrecientes. En las tres encuestas se presentaron grupos de puntos de interés, que incluían zonas en las que las regiones de Amhara, Afar y Tigray comparten vecindades.

Conclusiones: la distribución de la infección por VIH en Etiopía no es aleatoria en ninguna de las tres encuestas EDHS. Se observaron sistemáticamente grupos con una elevada concentración de casos de VIH en Addis Abeba y en las zonas vecinas de los estados regionales de Afar Tigray y Amhara, así como de la región de Oromia central. Este análisis reveló que aún hay áreas que deben seguir siendo estudiadas con respecto a la epidemia de VIH. En este sentido, Addis Abeba, ciertas áreas del estado regional de Amhara, grandes áreas de la región de Afar y Oromia central requieren atención especial.

Translated from English version into Spanish by María Luz Puerta, Revised by Mayra León, through

TRANSLATORS
WITHOUT BORDERS
